# Supplementary material for: The selection landscape and genetic legacy of ancient Eurasians
Source: Nature. 2024 Jan 10;625(7994):312–20. doi: 10.1038/s41586-023-06705-1 (PMC10781624; doi:10.1038/s41586-023-06705-1)
Supplement: Supplementary file 2 — Reporting Summary [file 41586_2023_6705_MOESM2_ESM.pdf]

nature portfolio

Corresponding author(s): Evan Irving-Pease, Rasmus Nielsen, Fernando Racimo, Eske Willerslev

Last updated by author(s): Sep 6, 2023

Reporting Summary

Nature Portfolio wishes to improve the reproducibility of the work that we publish. This form provides structure for consistency and transparency in reporting. For further information on Nature Portfolio policies, see our Editorial Policies and the Editorial Policy Checklist.

Statistics

For all statistical analyses, confirm that the following items are present in the figure legend, table legend, main text, or Methods section.

n/a	Confirmed	
☐	☒	The exact sample size (*n*) for each experimental group/condition, given as a discrete number and unit of measurement
☐	☒	A statement on whether measurements were taken from distinct samples or whether the same sample was measured repeatedly
☐	☒	The statistical test(s) used AND whether they are one- or two-sided<br>*Only common tests should be described solely by name; describe more complex techniques in the Methods section.*
☐	☒	A description of all covariates tested
☐	☒	A description of any assumptions or corrections, such as tests of normality and adjustment for multiple comparisons
☐	☒	A full description of the statistical parameters including central tendency (e.g. means) or other basic estimates (e.g. regression coefficient) AND variation (e.g. standard deviation) or associated estimates of uncertainty (e.g. confidence intervals)
☐	☒	For null hypothesis testing, the test statistic (e.g. *F*, *t*, *r*) with confidence intervals, effect sizes, degrees of freedom and *P* value noted<br>*Give P values as exact values whenever suitable.*
☐	☒	For Bayesian analysis, information on the choice of priors and Markov chain Monte Carlo settings
☐	☒	For hierarchical and complex designs, identification of the appropriate level for tests and full reporting of outcomes
☐	☒	Estimates of effect sizes (e.g. Cohen's *d*, Pearson's *r*), indicating how they were calculated

Our web collection on statistics for biologists contains articles on many of the points above.

Software and code

Policy information about availability of computer code

Data collection	No software was used for data collection.
Data analysis	The scripts used to run the chromosome painting (Supplementary Note 1b) and calculate ARS in the UK Biobank (Supplementary Note 2f) are available at https://github.com/will-camb/mesoneo_selection_paper (https://doi.org/10.5281/zenodo.8301166). The software to perform the ancestral path chromosome painting described in Supplementary Note 1c is available on GitHub at https://github.com/AliPearson/AncestralPaths (https://doi.org/10.5281/zenodo.8319452), and the demographic model is available in the stdpopsim library (see https://popsim-consortium.github.io/ stdpopsim-docs/stable/catalog.html#sec_catalog_homsap_models_ancienteurope_4a21). The analysis pipeline and ‘conda’ environment necessary to replicate the analysis of allele frequency trajectories of trait-associated variants in Supplementary Note 2a are available on Github at https://github.com/ekirving/mesoneo_paper (https://doi.org/10.5281/zenodo.8289755). The modified version of CLUES used in this study is available from https://github.com/standard-aaron/clues (https://doi.org/10.5281/zenodo.8228252). The pipeline to replicate the analyses for Supplementary Note 2d can be found at https://github.com/albarema/neo (https://doi.org/10.5281/zenodo.8301253). All other analyses relied upon available software which has been fully referenced in the manuscript and detailed in the relevant supplementary notes, including: bcftools v1.10.2, bedtools v2.29.2, biopython v1.76, clues v36cb7de, conda v4.9.0, numpy v1.17.0, pandas v1.0.4, pysam v0.15.3, python v3.6.7, r-base v3.6.1, r-bedr v1.0.7, r-dplyr v0.8.0.1, r-ggplot2 v3.1.1, r-ggrastr v0.2.1, r-ggrepel v0.8.2, r-ggridges v0.5.1, r-stringr v1.4.0, relate v1.1.3, scipy v1.4.1, and snakemake v5.12.3.

For manuscripts utilizing custom algorithms or software that are central to the research but not yet described in published literature, software must be made available to editors and reviewers. We strongly encourage code deposition in a community repository (e.g. GitHub). See the Nature Portfolio guidelines for submitting code & software for further information.

Data

Policy information about availability of data

All manuscripts must include a data availability statement. This statement should provide the following information, where applicable:

- Accession codes, unique identifiers, or web links for publicly available datasets
- A description of any restrictions on data availability
- For clinical datasets or third party data, please ensure that the statement adheres to our policy

All ancient genomic data used in this study are already published and listed in Supplementary Table S1. Data was aligned to the human reference GRCh37. Modern human genomes were obtained from the 1000 Genomes Project (1KGP), the Simons Genome Diversity Project (SGDP) and the Human Genome Diversity Project (HGDP). GWAS data was obtained from the GWAS Catalog, the FinnGen Study, and the UK Biobank (UKB).

Research involving human participants, their data, or biological material

Policy information about studies with human participants or human data. See also policy information about sex, gender (identity/presentation), and sexual orientation and race, ethnicity and racism.

Reporting on sex and gender	Sex was assigned based on sex chromosomes. Sex-specific results were not calculated.
Reporting on race, ethnicity, or other socially relevant groupings	Reporting was restricted to a self-identified 'white British' cohort, with PCA outliers removed (details in Bycroft et al., 2018).
Population characteristics	*Describe the covariate-relevant population characteristics of the human research participants (e.g. age, genotypic information, past and current diagnosis and treatment categories). If you filled out the behavioural & social sciences study design questions and have nothing to add here, write "See above."*
Recruitment	*Describe how participants were recruited. Outline any potential self-selection bias or other biases that may be present and how these are likely to impact results.*
Ethics oversight	Use of the UK Biobank resource was approved in 2020.

Note that full information on the approval of the study protocol must also be provided in the manuscript.

Field-specific reporting

Please select the one below that is the best fit for your research. If you are not sure, read the appropriate sections before making your selection.

☐ Life sciences ☐ Behavioural & social sciences ☒ Ecological, evolutionary & environmental sciences

For a reference copy of the document with all sections, see nature.com/documents/nr-reporting-summary-flat.pdf

Ecological, evolutionary & environmental sciences study design

All studies must disclose on these points even when the disclosure is negative.

Study description	Using an imputed dataset of >1600 complete ancient genome sequences, and new computational methods for locating selection in time and space, we reconstructed the selection landscape of the transition from hunting and gathering, to farming and pastoralism across West Eurasia.
Research sample	Our analyses are undertaken on a dataset comprising 1664 imputed diploid ancient genomes, and more than 8.5 million SNPs. These samples represent a considerable transect of Eurasia, ranging longitudinally from the Atlantic coast to Lake Baikal, and latitudinally from Scandinavia to the Middle East. Included are many of the key Mesolithic and Neolithic cultures of Western Eurasia, Ukraine, western Russia, and the Trans-Urals, constituting a thorough temporal sequence of human populations from 11,000 cal. BP to 3,000 cal. BP.
Sampling strategy	Sampling was dependent upon the availability of ancient human remains, though a considerable transect of the Mesolithic and Neolithic periods in Eurasia was represented, together with a detailed continuous sequence of human occupation of Denmark specifically. Our assemblage is well-represented with individuals of such key archaeological complexes as the Maglemose, Ertebølle and Funnel Beaker cultures in Scandinavia, the Cardial in the Mediterranean, the Körös and Linear Pottery complexes in SE and Central Europe, and many archaeological cultures in Ukraine, western Russia, and the trans-Ural (e.g. Veretye, Lyalovo, Volosovo, Kitoi).
Data collection	We collected 1664 previously published ancient shotgun genomes from 70 prior publications.
Timing and spatial scale	Sample chronology was generated by radiocarbon dating, with dates corrected for marine and freshwater reservoir effects. These showed samples ranging from the Upper Palaeolithic (c. 25,700 cal. BP) to the mediaeval period (c. 1200 cal. BP). Most individuals

	(97%, N=309) span 11,000 cal. BP to 3,000 cal. BP, the period broadly associated with the Mesolithic and Neolithic in Eurasia. Our research area can broadly be divided into three large regions: 1) central, western and northern Europe, 2) eastern Europe including western Russia and Ukraine, and 3) the Urals and western Siberia.
Data exclusions	Low coverage and related samples were excluded.
Reproducibility	Data quality and uncertainty (e.g. contamination) was accounted for in computational analyses to assess robustness of inferences, and all methods and data are made available for future replication.
Randomization	Sample groups were defined to reflect archaeological populations, based on phylogenetic inferences, temporal and geographic provenance, and cultural interpretations evidenced by archaeological contexts.
Blinding	Blinding was not applicable to this study.

Did the study involve field work? ☐ Yes ☒ No

Reporting for specific materials, systems and methods

We require information from authors about some types of materials, experimental systems and methods used in many studies. Here, indicate whether each material, system or method listed is relevant to your study. If you are not sure if a list item applies to your research, read the appropriate section before selecting a response.

Materials & experimental systems

n/a	Involved in the study	
☒	☐	Antibodies
☒	☐	Eukaryotic cell lines
☐	☒	Palaeontology and archaeology
☒	☐	Animals and other organisms
☒	☐	Clinical data
☒	☐	Dual use research of concern
☒	☐	Plants

Methods

n/a	Involved in the study	
☒	☐	ChIP-seq
☒	☐	Flow cytometry
☒	☐	MRI-based neuroimaging

Palaeontology and Archaeology

Specimen provenance	Details of the provenance of all samples analyzed in this study are available in Supplementary Table S1.
Specimen deposition	*Indicate where the specimens have been deposited to permit free access by other researchers.*
Dating methods	*If new dates are provided, describe how they were obtained (e.g. collection, storage, sample pretreatment and measurement), where they were obtained (i.e. lab name), the calibration program and the protocol for quality assurance OR state that no new dates are provided.*

☒ Tick this box to confirm that the raw and calibrated dates are available in the paper or in Supplementary Information.

Ethics oversight	No ethical approval required.

Note that full information on the approval of the study protocol must also be provided in the manuscript.
